# Guides and routes in a changing treatment landscape for immune-mediated neuropathies

**DOI:** 10.1186/s42466-026-00553-w

**Published:** 2026-11-12

**Authors:** Burkhard Gess

**Affiliations:** Universitätsklinik für Neurologie, Evangelisches Klinikum Bethel, Universität Bielefeld, Burgsteig 13, Bielefeld, 33617 Germany

## Main text

After a long time of relative standstill, the approval of efgartigimod-alfa for the treatment of CIDP has brought new momentum into the CIDP field. Efgartigimod-alfa was previously approved for the add-on treatment of generalized Myasthenia gravis with acetylcholine-receptor-antibodies [1]. It was then studied for CIDP in the ADHERE trial, showing efficacy in CIDP therapy and superiority to placebo over the course of 48 weeks [2]. Of note, only patients with evidence of active disease in a run-in period off-treatment were included in the study [2]. Only patients with confirmed evidence of improvement on open-label efgartigimod entered into the randomized phase of efgartigimod versus placebo [2]. In Germany, efgartigimod-alfa was approved for CIDP patients with prior treatment with IVIG or glucocorticoids. Interim analysis of a long-term follow-up study reported no significant increase in adverse events and prolonged clinically meaningful improvement [3]. Further long-term observations of efgartigimod-alfa in CIDP will follow.

In this issue of *Neurological Research and Practice* Sommer et al. present an S2e guideline summarizing the current recommendations for treatment of immune-mediated neuropathies, encompassing not only CIDP but also Guillain-Barré-Syndrome and diverse other immune-mediated neuropathies [4]. The authors conducted a systematic literature review, methodological quality assessment, risk of bias analysis and level of evidence classification, generating a guideline of high methodological quality.

Several changes in this up-to-date guideline are of particular relevance to clinical practice. One important aspect is the recognition of immune-mediated neuropathies associated with immune checkpoint inhibitors (ICIs). These neuropathies may develop with either a GBS-like or CIDP-like phenotype. Given the increasingly widespread use of ICIs in oncology, ICI-associated neuropathies should be considered in cancer patients presenting with compatible neurological symptoms. Treatment may include glucocorticoids, while modification or interruption of the underlying cancer therapy may be necessary in more severe cases [4].

With regard to CIDP, the guideline addresses several aspects of long-term treatment. Subcutaneous immunoglobulins (SCIg) are considered probably equivalent to intravenous immunoglobulins (IVIg) in maintenance therapy and therefore represent an alternative treatment option. Another important development is the introduction of efgartigimod, a neonatal Fc receptor (FcRn) inhibitor. Its efficacy has been demonstrated in a randomized controlled trial [2]. In Germany, efgartigimod is already approved as monotherapy for adults with progressive or relapsing active CIDP who have previously received corticosteroids or immunoglobulins.

For acute GBS, the guideline continues to emphasize the importance of timely immunotherapy. Both IVIg and plasma exchange are considered equally effective and superior to placebo. One particularly relevant change concerns patients who do not respond to initial IVIg treatment: a second course of IVIg is no longer recommended in this situation. The question remains which therapeutic course may be adopted for GBS patients who do not initially respond to IVIg treatment or apheresis therapy. Additional therapeutic agents for GBS represent an unmet clinical need [5].

In addition to the guideline and in parallel in this issue of *Neurological Research and Practice* Stettner et al. present a suggested treatment algorithm that includes diagnostic check-ups and re-evaluations of disease activity [6]. As CIDP is expected to improve under immunomodulatory therapy, the absence of such an improvement may prompt a diagnostic check-up. This is especially important since CIDP has many differential diagnoses, some of which are very rare and require additional testing – such as amyloidosis, hereditary neuropathy or peripheral nerve vasculitis [7]. On the other hand, CIDP may reach remission under therapy. Thus, routine check-ups of disease activity and treatment pauses are indicated to ascertain the need for further treatment. These issues were integrated into the treatment algorithm of Stettner et al. [6], albeit making it more complex and somewhat harder to appreciate at first glance. Nevertheless, this algorithm may be very useful to neurological practitioners for both in- and out-patient settings. The complex yet comprehensive design of the algorithm and flow chart make it useful for both general neurologists and neuro-muscular specialists.

Taken together, the guideline and the therapeutic algorithm offer both a comprehensive overview of evidence-based therapy for immune-mediated neuropathies and a practical hands-on treatment pathway for CIDP. Future developments will certainly necessitate additions and adaptations. Moreover, further research is necessary for CIDP, GBS and especially for rarer auto-immune neuropathies to improve evidence-based treatment. CIDP treatment is currently undergoing a period of rapid change. Thus, further developments and – hopefully – major therapeutic breakthroughs are to be expected. It is therefore highly encouraging that the German Neurological Society (DGN) is accompanying these developments with a guideline and a consensus paper, which may substantially facilitate the implementation of new treatment options in clinical practice.

## Data Availability

No datasets were generated or analysed during the current study.
